# The persistent impact of drought stress on the resilience of summer maize

**DOI:** 10.3389/fpls.2023.1016993

**Published:** 2023-01-25

**Authors:** Lanshu Jing, Baisha Weng, Denghua Yan, Shanjun Zhang, Wuxia Bi, Siying Yan

**Affiliations:** ^1^ State Key Laboratory of Simulation and Regulation of Water Cycle in River Basin, China Institute of Water Resources and Hydropower Research, Beijing, China; ^2^ College of Hydrology and Water Resources, Hohai University, Nanjing, China; ^3^ Yinshanbeilu Grassland Eco-hydrology National Observation and Research Station, China Institute of Water Resources and Hydropower Research, Beijing, China

**Keywords:** drought degree, drought duration, root growth, summer maize growth, PCA analysis

## Abstract

Crop resilience refers to the adaptive ability of crops to resist drought at a certain level. Currently, most of the research focuses on the changes in root or photosynthesis traits of crops after drought and rehydration. Still, the persistence effect (drought period (T2) - rehydration period (T3) - harvest period (T4)) of drought stress on crops and quantitative estimation of resilience is still unclear. Field experiments were conducted in this study to determine the persistence effects on above-ground and below-ground growth indicators of summer maize at different levels and durations of drought. Next, an evaluation method for integrated resilience of summer maize was proposed, and a quantitative assessment of integrated resilience was made by Principal Component Analysis (PCA) and resilience index calculation. The results showed that the resilience of summer maize decreased with increasing drought levels, which persisted until harvest. Although summer maize resilience was strong after rewatering under light drought (DR1), declined after sustained rewatering. At the same time, production had decreased. However, a specific drought duration could improve the resilience of summer maize under light drought conditions. In particular, leaf biomass and root growth in the 30-50 cm layer could be enhanced under long duration light drought (LDR1), thus improving summer maize resilience and yield. Thus, under water shortage conditions, a certain level and duration drought could improve the resilience and yield of summer maize, which would persist until harvest. Clarifying the persistent effects on the growth indicators of summer maize and quantitatively evaluating the resilience of summer maize could improve agricultural food production and water use efficiency.

## 1 Introduction

Under climate change, drought will become more frequent and severe in most parts of the world (Spinoni et al., 2020; Pokhrel et al., 2021; Li P, et al., 2022). As a kind of extreme weather phenomenon, drought is the main factor limiting agricultural production (Panda et al., 2021; Yao et al., 2022). Maize is one of the most significant food crops globally. Meanwhile, maize yield was more sensitive to drought (Liu et al., 2022). For instance, drought caused maize biomass losses in the northeast and north China plain from 2000 to 2019 (Cai et al., 2020; Wan et al., 2022). In the summer maize planting-tasseling and tasseling-physiological maturity periods, drought showed an increasing trend in the most Huang-Huai-Hai Plain of China from 1961 to 2015 (Wu X, et al., 2021). Consequently, drought poses a serious threat to maize growth and production.

The level and duration of droughts are closely related to maize growth. At the young ear development of maize, the maize biomass decreased by 54.3% and 61.4% under light (soil moisture at 60 ± 5%) and moderate (soil moisture at 40 ± 5%) drought stress, respectively (Shao et al., 2021). And as the drought increased in severity, the maize biomass and yield decreased (Zhang et al., 2019; Li et al., 2020; Liu et al., 2022). And, severe drought stress (35% field water capacity) adversely affected growth and yield parameters at different maize growth stages and resulted in delayed maturation (Ge et al., 2011). In addition, prolonged drought resulted in a reduction of growth rate, lower biomass, and delayed flowering (Verbraeken et al., 2021). For instance, with increasing drought duration, the number of filled grains per maize plant declined (Shin et al., 2015). Meanwhile, prolonged drought could cause maize cell dysfunction by the accumulation of reactive oxygen species (Xiao et al., 2020).

However, some researches also showed that during rehydration after drought, maize could recover, which was closely related to the degree and duration of drought (Ge et al., 2011; Sun et al., 2016). For example, drought limited the photosynthetic of maize, after rehydration could restore most photosynthetic traits (Qi et al., 2021). But the longer the drought duration, the weaker the maize’s photosynthetic capacity to recover (Jia et al., 2020). Although drought can affect the growth and development of maize, maize has resilience to resist drought. With different levels and durations of drought, maize resilience varies.

The resilience enables that crop could recover even exceed normal growth after rehydration at a certain level and duration drought (Müller et al., 2016; Qi et al., 2021). Originally, resilience refers to the ability of an ecological system to sustain relationships within an environmental system (Holling, 1973; Müller et al., 2016). Subsequently, some scholars proposed that resilience be incorporated into agroecosystems research, and believed that resilience could add tangible value to agroecosystems research (Peterson et al., 2018). Thus, some studies found crop diversity might contribute to increase crop resilience at a certain level drought (Elsalahy et al., 2020). Beyond that, coordinated changes in chlorophyll content, gas exchange, and fluorescence parameters possibly contributed to enhance maize resilience under drought conditions (Qi et al., 2021). Crop resilience is rapidly becoming a hot research issue. However, few studies have examined maize resilience under drought conditions based on maize growth indicators (Matsushita et al., 2016; El Chami et al., 2020).

Here, we conducted field experiments to quantitatively describe the persistent effects of drought on summer maize growth indexes and yield. Furthermore, we constructed the comprehensive resilience assessment method based on the growth indexes and quantitatively analyzed the resilience under different drought degrees and durations. This study aimed to test the following hypotheses: (і) Different degrees and durations of droughts can significantly reduce the resilience of summer maize. (іі) The drought impact on summer maize can last until the harvest period and then reduce the yield of summer maize. These results can contribute to better understanding the persistent impacts on summer maize resilience and serve as a scientific reference for drought risk responses in agriculture.

## 2 Materials and methods

### 2.1 Site description and drought evaluation

The experiment was conducted at Wudaogou experimental research station (33°09′N, 117°21′E) in Bengbu City, China. The area is a subtropical semi-humid warm temperate, continental monsoon climate. The main soil types are dark-hydromorphic clay loam and flavor-aquic soil, which account for 52.2% and 30.9% of the total area, respectively. The soil bulk density is 1.41 g/cm^3^ (Bi et al., 2021). Precipitation is unevenly distributed throughout the year, and droughts and floods are frequent. It is the central disaster area of agricultural drought in the Huaihe River Basin (Liu et al., 2017; Chen et al., 2020; Wang et al., 2020).

The drought degree is determined by China’s “Standards of Drought Situations” (SL424-2008). The calculation equation was as follows:


$$\text{RSH}=\frac{\theta }{{\text{F}}_{\text{c}}}\times 100\%$$

Where ***θ*** was the soil average weight moisture, which was calculated from the ratio of soil volumetric water content to soil bulk density in 10-20 cm layer. ***F_c_*** was the field capacity (Bi et al., 2021).

The drought is divided into five levels. When RSH>60% means no drought; When RSH is within 50%-60%, indicating light drought; When RSH is within 40%-50%, indicating moderate drought; When RSH is within 30%-40%, indicating severe drought; When RSH< 30%, indicating extreme drought.

### 2.2 Experiment design

**Figure 1:**
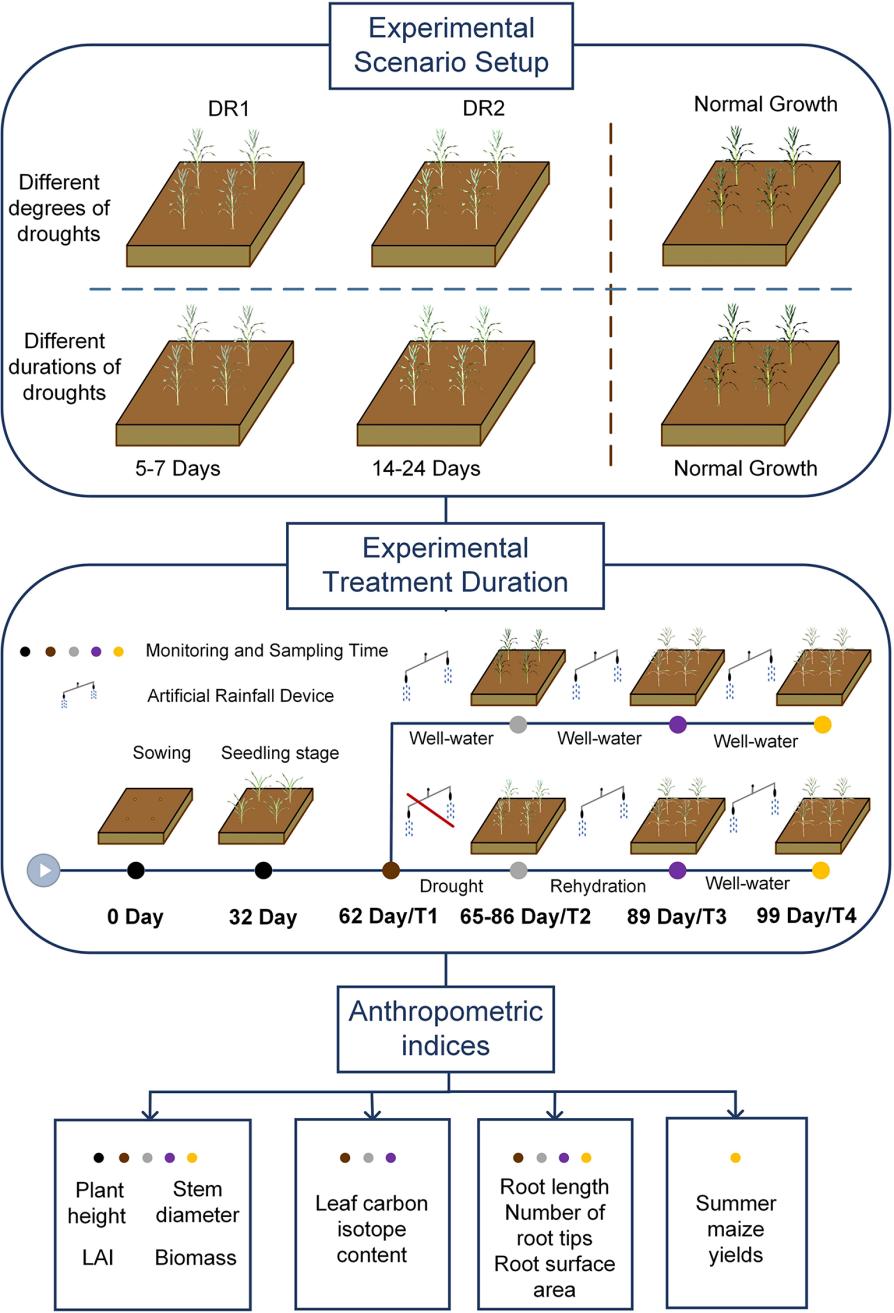
The specific process of the experiment. DR1, Light drought; DR2, Moderate drought; T1, before drought; T2, drought period; T3, rehydration period; T4, harvest period.

We explored the drought persistent impact on the resilience of summer maize during jointing, tasseling, and grain filling. We designed six drought scenarios, i.e., Light drought (DR1), Moderate drought (DR2), Short duration light drought (5 days, SDR1), Long duration light drought (7 days, LDR1), Short duration moderate drought (14 days, SDR2), and long duration moderate drought (24 days, LDR2). In addition, a control system (CK) was set for reference under no drought conditions. The specific process of the experiment is shown in **Figure 1**. In particular, before the start of the experiment, the initial conditions of each field were the same (seed type, fertilization amount, suitable soil water content). And the ventilated shed with an artificial rainfall device was used to block the entry of external precipitation. After the drought, the soil moisture would be restored to the suitable summer maize growth range through artificial rainfall.

**Figure 2:**
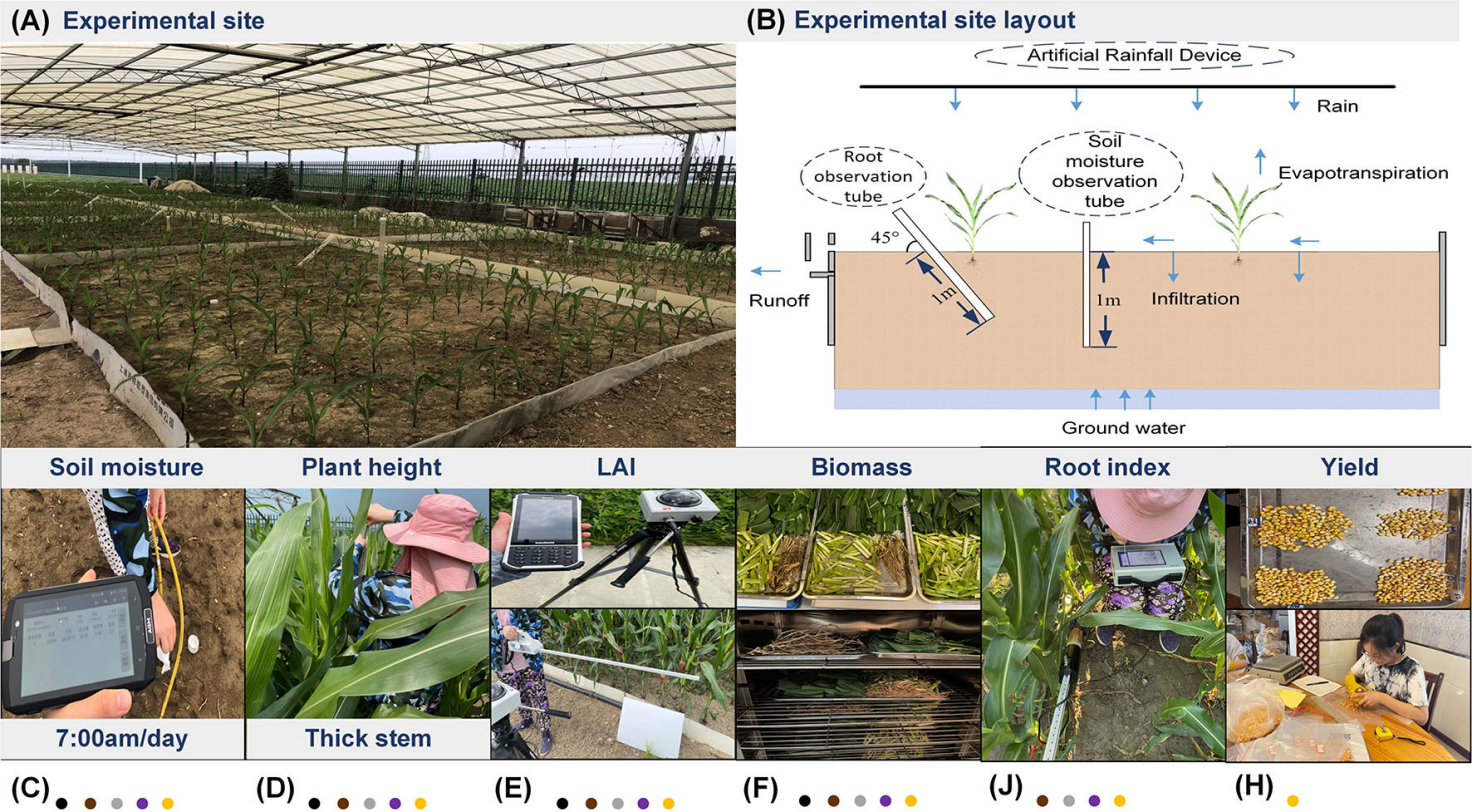
**(A)** experimental site and **(B)** experimental site layout and **(C)** soil moisture monitoring and **(D)** plant height and thick stem monitoring and **(E)** LAI monitoring and **(F)** biomass monitoring and **(J)** root monitoring and **(H)** yield monitoring.

All experiments were conducted during the growing season of summer maize from 18 June to 28 September 2020 and from 19 June to 25 September 2021. Field planting was used at the experiment site. Due to the limitations of the site, each scenario included two fields (length×width=3.7 m×5.5 m). **Figures 2A, B** shows the specific layout details. Each area used aluminum-plastic composite panels with a depth of 1.2 m as baffles to prevent shallow water and groundwater. Apart from this, Denghai 618 was selected as the sowing variety of summer maize in the experiment, and the fertilizer was urea and compound fertilizer. The row spacing is 60 cm and the plant spacing is 25 cm, and about 135 plants were planted in each field.

### 2.3 Soil moisture measurement

Soil moisture was measured with an AIM-WiFi soil multiparameter monitoring system (Beijing Aozuo Ecology Instrumentation Ltd., Beijing, China), which used the Time Domain Reflectometry principle (**Figure 2C**). The rated accuracy was 1%. In this experiment, we collected four layers of soil moisture data (10 cm, 30 cm, 50 cm, and 70 cm) in each field from 7:00 am to 8:00 am (GMT + 8), and each layer was measured three times. This was one of the primary indicators to judge the levels of drought.

### 2.4 Growth index measurement

The growth indexes included plant height, thick stem, leaf area index (LAI), biomass, leaf carbon isotope content, root surface area, root length, number of root tips, and summer maize yield. Notably, plant height and thick stem were measured with a tape measure (**Figure 2D**), and each field selected three plants for measurement. LAI was measured with a Sun-scan system (Senpro Mechanical & Electrical Co., Ltd, Shanghai, China) (**Figure 2E**). To ensure the accuracy of the measurement results, each measurement time was selected from 9:00 am to 11:00 am (GMT + 8) in the morning. Biomass data (root, stem, leaf, fruit) were obtained by digging the whole summer maize, decomposing, drying, and weighing it (**Figure 2F**). The root growth indexes (the root surface area (RSA), root tip number (RNB), and root length (RLT)) were measured by the micro-root window technology of the AZR_100 root ecological monitoring system (Beijing Aozuo Ecology Instrumentation Ltd., Beijing, China) (**Figure 2G**). Each point measured 4 layers, 10 cm, 30 cm, 50 cm and 70 cm. The yield of summer maize was determined by the statistics of agronomic traits of mature maize ears in each field. (**Figure 2H**).

### 2.5 Calculation of comprehensive resilience index

Based on field experiments, we used the comprehensive resilience index to quantitatively analyze the persistence effects of different drought on the resilience of summer maize. To establish a comprehensive resilience index, we need to clarify the contribution rate of each growth index of summer maize under drought conditions. The purpose of principal component analysis (PCA) is to decrease the elements of the indicator dataset and to select new significant underlying factors (Banerjee et al., 2015; Otitoju et al., 2016). Therefore, we used the PCA method to select the central component (PC) and factor as the weight value of each main growth index.

In order to calculate the comprehensive resilience index, we also need to build a single factor resilience index, the specific formula is as follows (Orwin and Wardle, 2004; Elsalahy et al., 2020) (Eq (1)) :


$${\text{R}}_{\text{l}}=\{\begin{array}{c} 1-\frac{2\left|{\text{D}}_{0}\right|}{\left(\left|{\text{D}}_{0}\right|+\left|{\text{D}}_{\text{x}}\right|\right)},{\text{D}}_{\text{x}}>0\\ 0\;,\;{\text{D}}_{\text{x}}<0 \end{array}$$

Where ***D_0_*** is the difference between the indexes of no drought-stressed summer maize and drought-stressed summer maize at the end of drought; ***D_x_*** is the difference between drought-stressed summer maize and drought-stressed summer maize after rehydration. If ***D_x_* < 0,** it means that the summer maize failed to recover after rehydration (***R_l_*** = **0**); While ***D_x_* > 0,** the summer maize has resilience after rehydration. If ***R_l_* < 0**, indicates that summer maize has not been able to recover fully, the greater the ***R_l_***, the stronger the resilience; If ***R_l_*** ≥ **0**, indicates that summer maize has been able to recover fully, the greater the ***R_l_***, the stronger the resilience.

Further, according to the weight of each main growth index and single factor resilience, the comprehensive resilience index of summer maize is calculated. The formula is as follows: (Eq (2)):


$${\text{R}}_{\text{Tl}}=\lambda_{1}\times {\text{R}}_{\text{l}1}+\lambda_{2}\times {\text{R}}_{\text{l}2}+\cdot \cdot \cdot +\lambda_{\text{k}}\times {\text{R}}_{\text{lk}}$$

Where ***λ*_1_** … ***λ*_k_** are the weight of each main growth index (based on PCA calculation results); ***R_l1_*** … ***R_lk_*** are the resilience of main growth index.

### 2.6 Statistical analysis

The growth characteristics of summer maize under different drought conditions were analyzed by taking the difference between the drought-stressed and no drought-stressed summer maize indexes. The drought treatment group data minus the mean value of the control group data was used to represent the variation of each index. Difference data were shown in mean + standard deviation. The figures were drawn in Origin version 2021 (OriginLab Inc., Hampton, MA, USA).

## 3 Results

### 3.1 Effects of different drought degrees and durations on summer maize in the T2-T3 period

**Figure 3:**
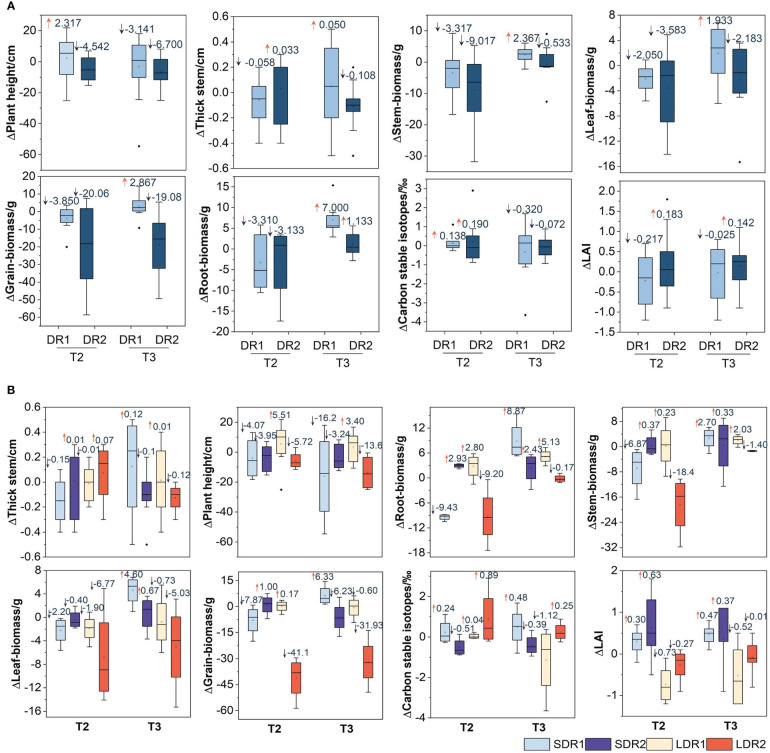
Effects of different **(A)** drought degree and **(B)** drought duration in the T2-T3 period on growth indexes of summer maize. in filling. SDR1, Short duration light drought (5 days); LDR1, Long duration light drought (7 days); SDR2, Short duration moderate drought (14 days); LDR2, Long-duration moderate drought (24 days).

**Figure 3A** showed that the changes of various growth indexes of maize in the T2-T3 period. In the T2 period, the biomass of stem, leaf, and grain decreased by 3.317 g, 2.05 g, and 3.85 g under DR1, 9.017 g, 3.583 g, and 20.06 g under DR2 respectively. DR2 decreased more than DR1. However, the reduction of root biomass under DR2 was less than that under DR1, which was 3.31 g and 3.133 g, respectively. In addition, the plant height under DR1 was taller than the CK, which was 2.317 cm. thick stem, carbon stable isotope, and LAI under DR2 were higher than that under DR1. Plant height, stem, leaf, and grain biomass decreased with drought degree. Meanwhile, thick stem, root biomass, carbon stable isotopes, and LAI increased slightly with the increasing of drought degree. In the T3 period (**Figure 3A**), the plant height, thick stem, stem, leaf, grain, and root biomass of summer maize affected by DR2 were lower than those affected by DR1. Additionally, LAI was lower than CK under DR1, but higher than CK under DR2. The thick stem, stem, leaf, grain, and root biomass affected by DR1 could be recovered to normal growth state, however, plant height, carbon stable isotopes, and LAI could not be recovered. While root biomass and LAI could recover to normal growth from DR2, other growth indicators are not. It represented that the stronger the drought level, the stronger the effect on summer maize growth indicators.

**Figure 4:**
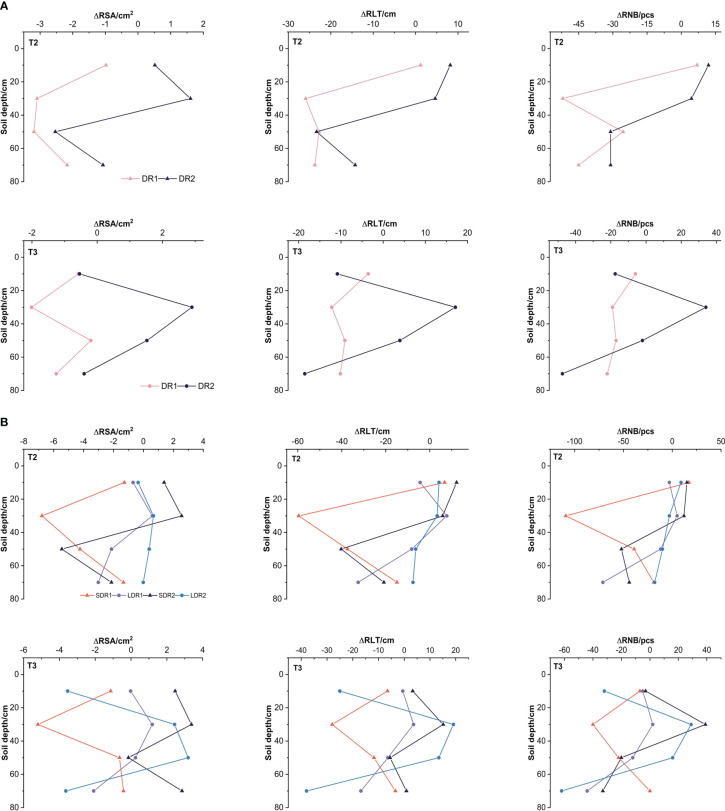
Effects of different **(A)** drought degree and **(B)** drought duration in the T2-T3 period on root growth indexes of summer maize. RSA, root surface area; RNB, root apex number; RLT, root length.

**Figure 4A** showed that the changes of various root growth indexes of maize in the T2-T3 period. In the T2 period, the reduction of root growth indexes under DR1 were higher than that under DR2, but only the reduction of the RNB and RLT in the 50 cm layer was lower. RSA in the 10 cm layer and RSA, RNB, and RLT in the 30 cm layer under DR2 were better than the normal growth state. The RSA, RNB, and RLT in the 50cm and 70cm layer under DR2 were worse than the normal growth state. In the T3 period (**Figure 4A**), the reduction of RSA, RNB, and RLT in the 10 cm and 70 cm layers under DR2 were higher than that under DR1, but only the reduction of the RSA in the 10 cm layer was lower. The RSA, RNB, and RLT in the 30-50 cm layer under DR2 were higher than that under DR1. The root growth of 10 cm and 70 cm was damaged and could not be recovered to the normal growth state. The RSA, RNB, and RLT in the 30-50 cm layer could recover to the normal growth state under DR2.

**Figure 3B** showed that the changes of various growth indexes of maize under different drought duration in the T2-T3 period. In the T2 period, thick stem, plant height, root, leaf, stem, grain biomass under LDR1 were all higher than that under SDR1. Carbon stable isotopes and LAI under SDR1 were higher than that under LDR1. plant height, root, stem, leaf, grain biomass, and LAI under SDR2 were all higher than that under LDR2. The thick stem and carbon stable isotope under SDR2 were lower than that under LDR2. In the T3 (**Figure 3B**), thick stem, root, stem, leaf, grain biomass, carbon stable isotope, and LAI under SDR1 were all higher than that under LDR1. The plant height under SDR1 was lower than that under LDR1. The plant height, thick stem, root, stem, leaf, grain biomass, and LAI under SDR2 were all higher than that under LDR2. The carbon stable isotope under SDR2 was lower than that under LDR2. In addition, the plant height could not recover to the normal growth state, other growth indicators could recover under SDR1. The thick stem, plant height, root, and stem biomass could recover to the normal growth state, other growth indicators could not recover under LDR1. The thick stem, plant height, grain biomass, and LAI could not recover to the normal growth state, other growth indicators could recover under SDR2. The carbon stable isotope could recover to the normal growth state, other growth indicators could not recover under LDR2.

**Figure 4B** showed that the changes of various root growth indexes of maize under different drought duration in the T2-T3 period. In the T2 period, the RSA, RNB, and RLT in the 30 and 50 cm layers were all higher under LDR1 than that under SDR1. However, the RSA, RNB, and RLT in the 10cm and 70 cm layers were all lower under LDR1 than under SDR1, except for the RSA in the 10 cm layer. The RSA, RNB, and RLT in the 10 cm and 30 cm layers under SDR2 were also lower than that under LDR2. The RSA, RNB, and RLT in the 50cm and 70cm layers under LDR2 were lower than that under SDR2. In the T3 period, the RSA, RNB, and RLT in the 10, 30, and 50 cm layers under LDR1 were all higher than that under SDR1. But the RSA, RNB, and RLT in the 70 cm layer under LDR1 was lower than that under SDR1. It indicated that under light drought stress, the root growth in the 10, 30, and 50 cm layers increased with the drought duration. Besides, the RSA, RNB, and RLT in the 10 cm, 30 cm, and 70 cm layers under SDR2 were higher than that under LDR2, apart from the RLT in the 30 cm. But the RSA, RNB, and RLT in the 50 cm under SDR2 were lower than that under LDR2. It could be explained that under moderate drought stress, the root growth in the 10, 30, and 70 cm layers decreased with the drought duration.

### 3.2 Effects of different drought degrees and durations on summer maize in the T4 period (harvest period)

**Figure 5:**
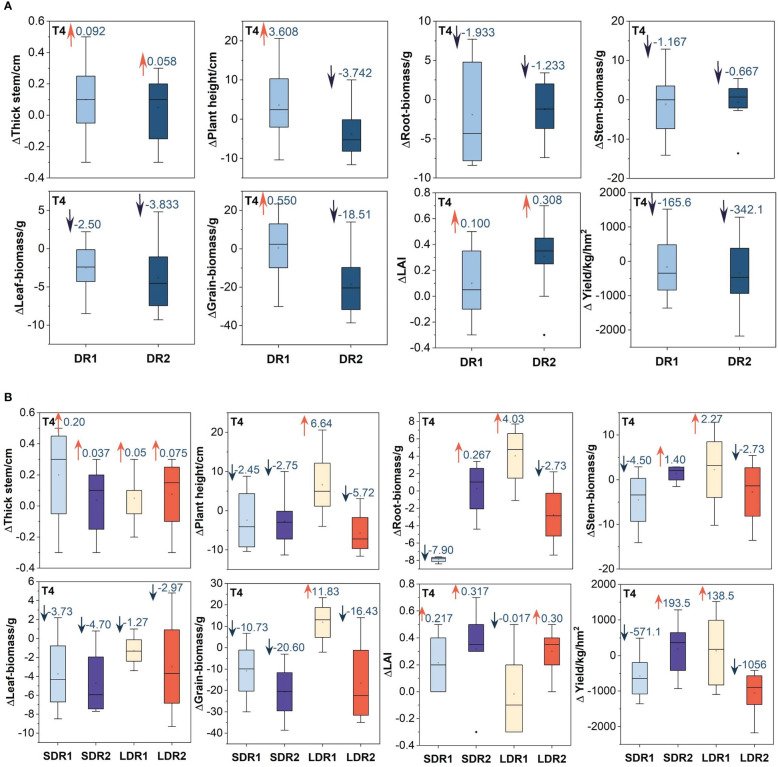
The persistent effects of different **(A)** drought degrees and **(B)** drought duration on growth indexes of summer maize in the T4 period.

The change in each growth indexes in the T4 period could reflect the persistent effect of drought on the growth process of summer maize. **Figure 5A** showed that in the T4 period, root, stem, leaf biomass, and yield were lower than CK. Although the root, stem, leaf, and grain biomass affected by drought in the T3 period could recover to the normal growth state, but it could not last until harvest. Plant height and grain biomass under DR1 were higher than CK, 3.608 cm and 0.55 g, respectively. But, that under DR2 were lower than CK, 3.742 cm and 18.51 g, respectively. It points out that the recovery of summer maize could decreased with the drought degree. In addition, the yield of summer maize decreased with the increase in drought degree.

**Figure 5B** showed that leaf biomass and LAI under LDR1 were lower than CK, but other growth indexes were higher than CK. The thick stem and LAI under SDR1 were higher than CK, but other growth indexes were lower than CK. However, compared with the T3 period, under light drought stress, the recovery and yield of summer maize could increase with the increased drought duration. Thick stem, root, stem biomass, LAI, and yield were higher than the CK under SDR2. Among them, except for thick stem and LAI, the other indicators under LDR2 were lower than CK. Therefore, compared with the T3 period, under moderate drought stress, the recovery and yield of summer maize could decrease with the increased drought duration.

**Figure 6:**
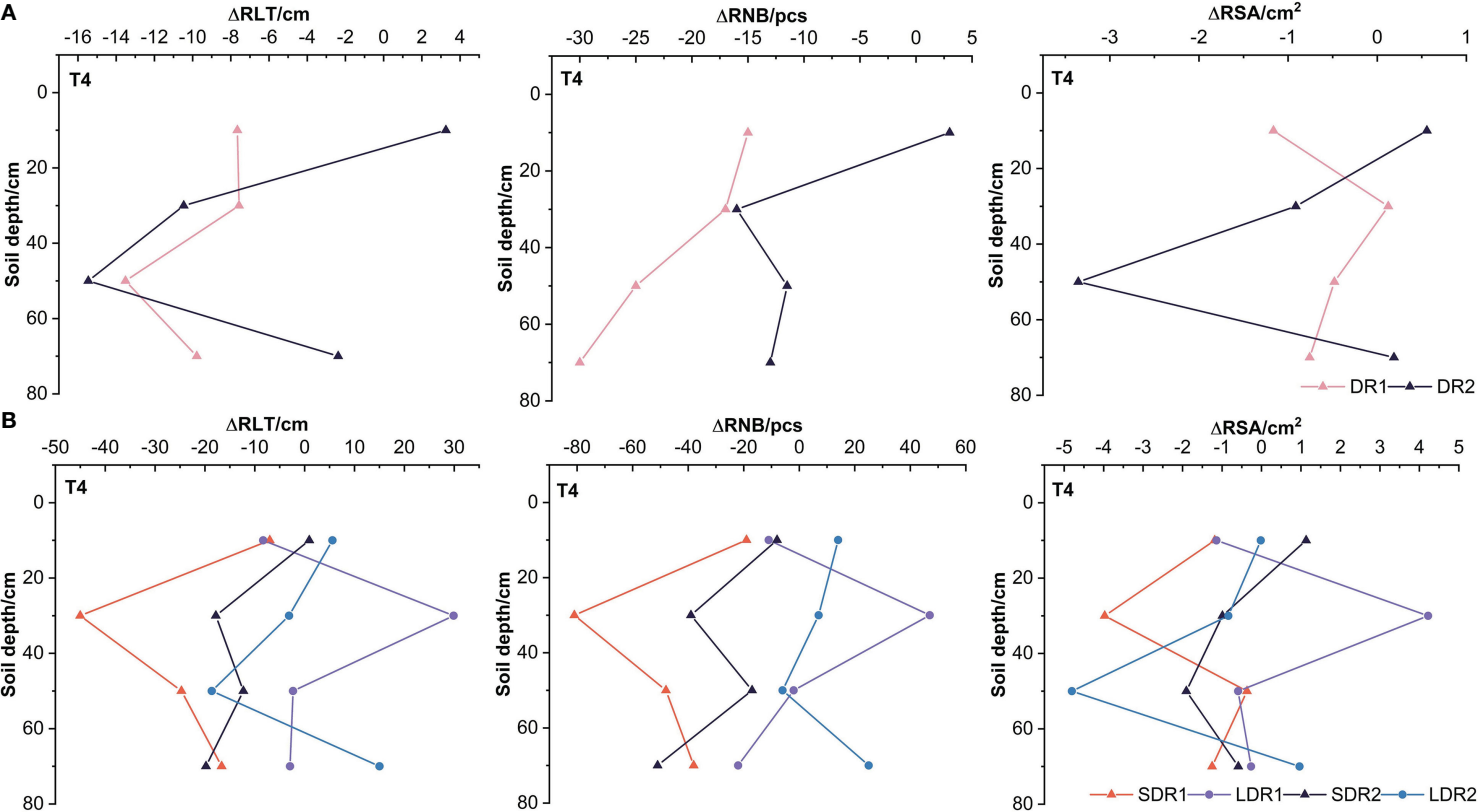
The persistent effects of different **(A)** drought degrees and **(B)** drought duration on root growth indexes of summer maize in the T4 period.

**Figure 6A** showed that the RSA, RNB, and RLT in the 10 cm layer and the RSA in the 70 cm layer under DR2 were higher than CK, but other conditions were lower than CK. Only the RSA in the 30 cm layer under DR1 was higher than CK, and other root indexes were lower than CK. Furthermore, the RSA and RLT in the 30 and 50 cm layer under DR2 were reduced more than that under the DR1. Compared with the T3 period, it indicated that drought reduced the root growth ability of summer maize in 30 and 50 cm layer, which increased with increasing drought levels in the T4 period. **Figure 6B** showed that the RSA, RNB, and RLT in the 30 cm layers were all higher than CK under LDR1. However, the RSA, RNB, and RLT in each layer were all lower than CK. Except for the RLT in the 10 cm and the RSA in the 50 cm, other root indexes under LDR1 were all higher than SDR1. Compared with the T3 period, it indicated that a certain duration of light drought could promote root growth and this effect can last until harvest in the T4 period. Apart from that, the RNB and RLT in the 10 cm layer, the RNB in the 30 cm layers and the RSA, RNB, and RLT in the 70 cm layer under LDR2 were higher than CK. The RSA, RNB, and RLT in the 30 cm, 70 cm layers and the RNB in the 50 cm layer under LDR2 were higher than SDR2. However, the reduction of RNB and RLT in the 30 cm layer were more elevated under LDR2 than SDR2. Thus, in the T4 period, under moderate drought stress, longer duration of drought significantly reduced root growth in the 30 cm layer and inhibited summer maize recovery.

### 3.3 The persistence effects of different drought degrees and durations on the resilience of summer maize

**Figure 7:**
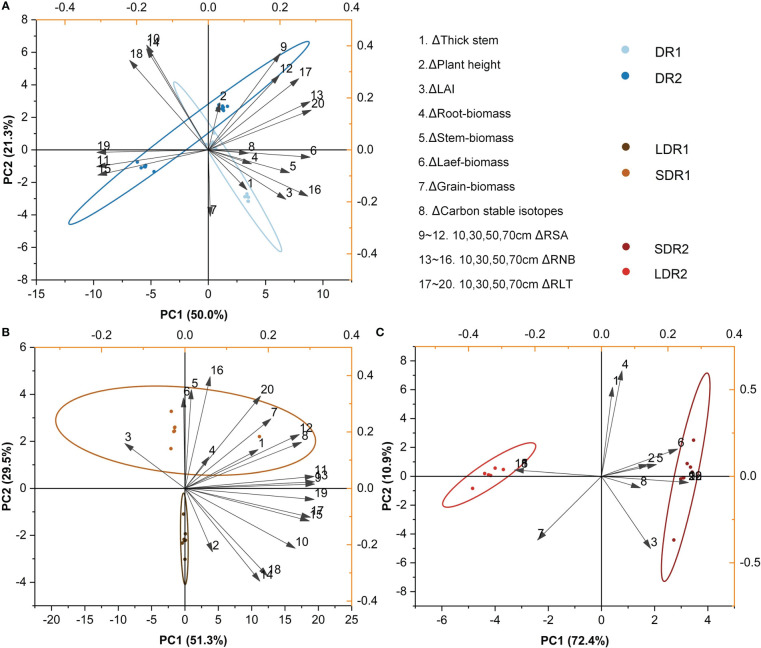
PCA analysis of the effects of different **(A)** drought degrees and **(B)** drought durations under light drought stress and **(C)** drought durations under moderate drought stress on various growth indexes of summer maize in the T3 period.

The main factors influencing summer maize growth during T3 were analyzed by PCA. **Figure 7A** showed that the main factors affecting summer maize growth under different degrees of drought were leaf biomass, carbon stable isotopes, RLT in the 50 cm layer and RSA in the 50 cm layer. **Figure 7B** showed that the main factors affecting summer maize growth under different durations of light drought stress were RSA in the 10 and 50 cm layers, RNB in the 10 cm layer and RLT. **Figure 7C** showed that under moderate drought stress, the main factors affecting summer maize growth at different durations were RSA, RNB and RLT in the 10-70 cm layer.

**Figure 8:**
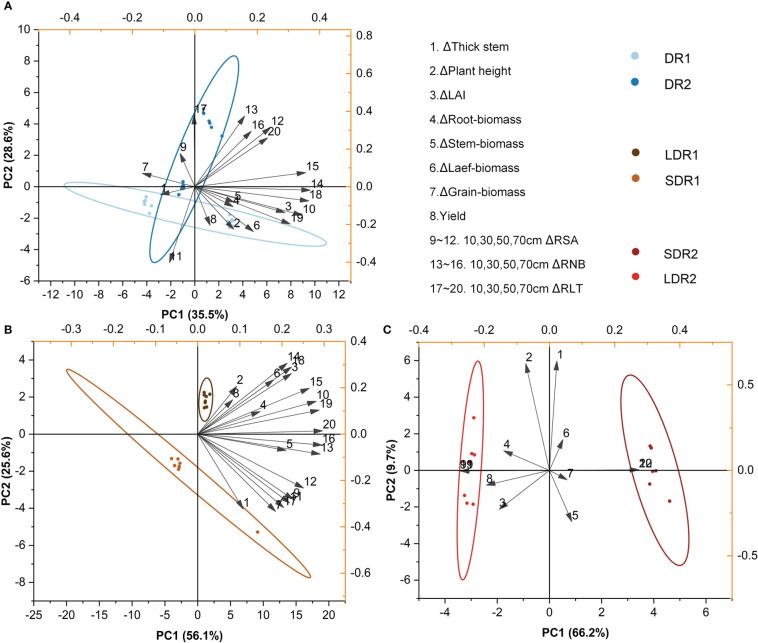
PCA analysis of the effects of different **(A)** drought degrees and **(B)** drought durations under light drought stress and **(C)** drought durations under moderate drought stress on various growth indexes of summer maize in the T4 period.

In the T4 period, **Figure 8A** showed that the main factors affecting persistence of summer maize at different drought levels were stem thickness, RNB and RLT in the 30 cm layer and grain biomass. **Figure 8B** showed that the main factors affecting persistence of summer maize under light drought stress at different durations were stem biomass, RNB in the 10 cm layer and RNA B and RLT in the 70 cm layer. **Figure 8C** showed that the main factors affecting summer maize under moderate drought stress were grain biomass, yield and RSA, RNB and RLT.

**Figure 9:**
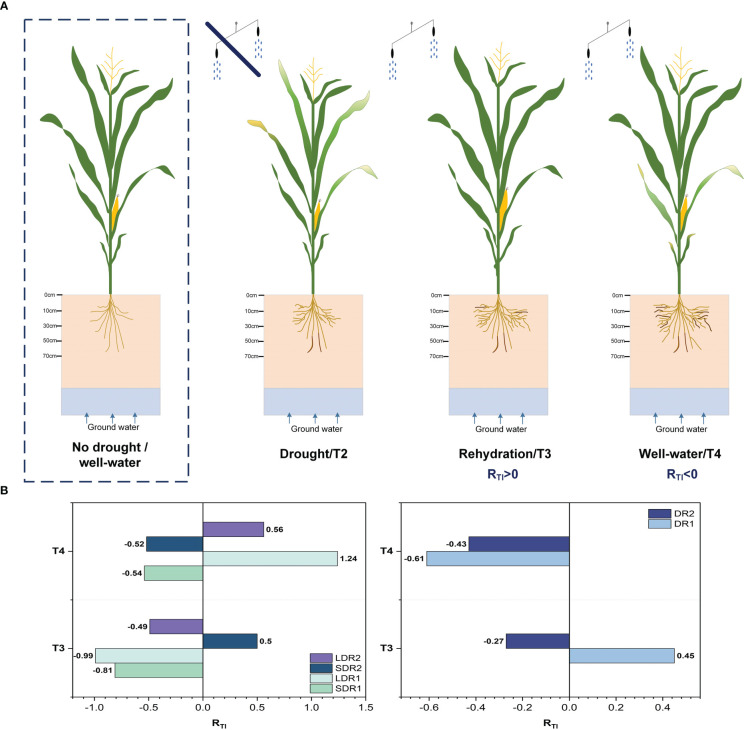
**(A)** Growth process and **(B)** resilience evaluation results of summer maize. (Darker color roots were dead roots, while lighter color roots were living roots.).

Based on these results, the resilience of summer maize under different drought stresses was quantified. **Figure 9** showed that drought-affected summer maize had two states of complete and incomplete resilience after rehydration. Thus, the resilience under DR2 was weaker than DR1 during T3. Moreover, summer maize could not recover completely under DR2, but could recover completely under DR1. In the T4 period, summer maize could not recover completely under both DR1 and DR2. However, the resilience under DR2 was higher than that under DR1. This revealed that the stronger the drought stress, the weaker the resilience in the T3 period. Meanwhile, the effect of drought on summer maize growth could last until the T4 period, reducing the resilience of summer maize. In addition, summer maize could not fully recover under SDR1 and LDR1 during T3 period, and the resilience under SDR1 was higher than that under LDR1. However, in T4 period, summer maize resilience increased under both drought conditions. And summer maize could recover completely under LDR1. This demonstrated that the resilience of long-term light drought was higher than that of short-term light drought under light drought stress. In the T3 period, summer maize has stronger resilience under SDR2 than LDR2, and summer maize could recover completely under SDR2 but not under LDR2. This suggest that summer maize affected by drought stress needs a certain amount of time to recover and a certain level of drought stress can enhance the resilience of summer maize.

## 4 Discussion

### 4.1 The response and resilience mechanism of summer maize under different drought degrees

Drought is a key factor affecting summer maize growth (Webber et al., 2018; Pokhrel et al., 2021). Previous studies had shown that increased drought limited the total biomass of summer maize plants, particularly grain and leaf biomass (Lesk et al., 2016; Shao et al., 2021; Wu H, et al., 2021). Moreover, drought stress reduced leaf size, stem elongation, and root proliferation, disrupted plant water relationships, and reduced water use efficiency (Efeoğlu et al., 2009; Farooq et al., 2009; Zhang et al., 2015). These conclusions were similar to those obtained in the T2 period analysis in this study. Drought stress could promote lateral root development, and induce lateral roots to grow into the middle soil layer to absorb more water, thereby increasing root biomass (Hussain et al., 2020; Hazman & Kabil, 2022). Also, this study showed that RSA, RLT and RNB in the 30 cm layer increased with increasing drought. A strong root structure could enhance the drought tolerance of maize (Hussain et al., 2020). Summer maize roots are affected by drought stress and secreted ABA (abscisic acid) synthase, which transmit drought signals to the aboveground organs (Wang et al., 2018). After receiving the drought signal, the leaves control the stomatal conductance through osmoregulation and reduce the photosynthetic rate and sap flow rate, thus reducing the biomass of each organ (Cai et al., 2020). Drought stress can also significantly alter the metabolism of carbohydrates, sugars and their derivatives, thereby reducing pollen viability and inhibiting the development of the male and fruiting ears (Li H, et al., 2022). This is in general agreement with the findings of the present study. However, this study still found that carbon stable isotopes and LAI increased slightly with increasing drought. The increase in carbon stable isotopes was mainly due to changes in enzyme activity in the leaves, leading to assimilation of C^13^ (Zhang et al., 2015). These phenomena indicated that drought stress negatively affected the growth of summer maize, but summer maize could resist certain drought damage.

However, summer maize affected by drought stress could recover after rehydrated (Qi et al., 2021). Previous studies had shown that maize could recover biomass, most photosynthetic traits, and growth rate, but not the plant height and leaf area (Efeoğlu et al., 2009; Song et al., 2018). The above results were only consistent with the summer maize recovery of DR1 in this study. This ability of summer maize was known as resilience. The resilience of summer maize to drought may be due to root-induced cytokines, which promote the recovery of summer maize growth after rehydration (Qin & Wang, 2018). And the overexpression of ZmbZIP33 could also promote the accumulation of abscisic acid and improve the resilience of summer maize (Cao et al., 2021). In addition, this study also found that the resilience of RSA, RNB and RLT in the 30-50 cm layer showed an increasing trend with increasing drought level. It indicates that root growth in the 30-50 cm layer plays an important role in the resilience of summer maize. Moreover, root hydraulic conductance was closely related to stomatal conductance (Wang et al., 2017). Photosynthetic and transpiration rates recovered gradually after root growth was restored (Shao et al., 2021). Sap rate recovered, but decreased with increasing drought (Cai et al., 2020). Similar findings were found in the present study, with the exception of root biomass and LAI, which decreased in resilience as the degree of drought increased. In addition, maize is also able to return to normal levels at different rates through energy production, osmotic protection and signal regulation, but there are some differences between the metabolome and normal levels, e.g., the anthocyanin and proline content of leaves decreases with increasing drought levels after rehydration. In contrast, sucrose and glucose are overcompensated after rehydration (Efeoğlu et al., 2009). And with the increase in the degree of drought, the photosynthesis ability weakened, the leaves senesced, and the biomass accumulation and transport ability of maize weakened (Mansouri-Far et al., 2010; Zhang et al., 2019). Further explanation, the resilience of summer maize decreased with the increase of drought level.

### 4.2 The response and resilience mechanism of summer maize under different drought duration

Drought duration was another key factor affecting summer maize growth (Qi et al., 2021). As previous studies have revealed that the long duration of drought effects on the process of plant growth, leaf morphological characteristics and photosynthesis were severely remarked (Song et al., 2018; Jia et al., 2020). The prolonged drought resulted in a reduction in growth rate of individual organs and an extension of growth duration of summer maize (Verbraeken et al., 2021). It was consistent with the results in this study, but the results obtained under light drought stress differently. This study found that longer duration of drought could promote the growth of summer maize under light drought stress. But carbon stable isotopes decreased with increasing drought duration. Longer duration of drought inhibited the growth of summer maize under moderate drought stress. The possible reason for this is that under mild drought, summer maize adapts to drought by changing enzyme activities in the root zone and leaves to improve water use efficiency (Hu et al., 2010). This revealed that the growth of the above-ground organs of summer maize is closely related to the root growth. Therefore, this study further analyzed the growth characteristics of the root system. Under light long-duration drought stress, summer maize improved its drought resistance by increasing and refining the root system in the 30-50 cm layer. As the drought level increased, the resistance of summer maize decreased. Under moderate short drought stress, summer maize improved resistance by increasing the root system in the 30 cm layer, whereas under moderate long-duration drought stress, summer maize improved resistance by increasing the root system in the 50 cm layer. But this ability was weaker than under moderate short-duration. This phenomenon may suggest that root growth in the 30-50 cm layer has a certain influence on the drought resistance of summer maize, but its ability is limited.

The recovery of summer maize affected by different drought durations after rewatering was different. This study found that under light and moderate drought stress, the recovery of summer maize growth was inhibited with the increase of drought duration. This result was consistent with some previous studies (Sun et al., 2016; Song et al., 2019; Jia et al., 2020). Ammonia-oxidizing bacterial strains in rhizosphere soil coexisted with maize and increased resilience by regulating soil nitrification and root-induced leaf cytokinin (Wang et al., 2022). The direct induction of cytokinin synthesis in maize roots by NO^3^ released from the soil due to inter-root soil nitrification (Wang et al., 2021). Cytokinin transport to the leaves promotes recovery of summer maize (Wang et al., 2018). The root is the most direct organ that receives the stimulation. The root structure of summer maize in the 30-50 cm layer played an important role in adapting to drought, while the root structure of summer maize in the layer of 10 cm could adapt to the influence of light drought, and the root structure of summer maize in the 70 cm layer was more seriously damaged during the drought and had weaker resilience.

### 4.3 The persistent effects of the resilience of summer maize under different degrees and durations of drought

Although many studies related to the effects of drought and rehydration conditions on physiological traits of summer maize, less attention paid to the effects of persistence on growth characteristics and yield of summer maize after rehydration at maturity (Sheoran et al., 2022). In this study, by analyzing the growth characteristics of summer maize during T4, we inferred that the effects of drought stress on summer maize continued until harvest, which was able to reduce the recovery capacity of summer maize and affect the yield. Also, the degree of root recovery of summer maize in the 30-50 cm layer decreased as the degree of drought increased. The root system is very closely related to summer maize resilience, indicating that summer maize does not sustain recovery. Furthermore, summer maize affected by severe drought, although it was able to recover to normal conditions after rehydration, continued rehydration still resulted in reduced growth status and lower yields.

It is worth noting that the effect of different drought durations on summer maize resilience during the T4 period was different. In this study, it was inferred that under light drought stress, a certain range of drought ephemeris and sustained rehydration increased the recovery and yield of summer maize, e.g., under LDR1. Although summer maize recovered to a lesser extent after rehydration, the resilience gradually increased. Yield was relatively higher after sustained rehydration. Under moderate drought stress, yields decreased with increasing drought duration, and the degree of recovery was higher but still below normal growth. Previous studies have shown that under drought stress, maize yield showed a non-linear response to drought severity (Jin et al., 2020; Shao et al., 2021; Liu et al., 2022). Consistent with the results of this study, summer maize yield could be increased under SDR2 conditions. This may be related to the root system. The root system in the 30 cm layer was less redacted and improved the resilience of summer maize under SDR2. Under water deficit conditions, a range of drought duration and continuous rehydration were able to improve summer maize resilience and yield.

### 4.4 Quantitative evaluation of resilience of summer maize

Roots and leaves played a key role in the growth process of summer maize under drought stress. Similar conclusions were obtained by PCA analysis in this study. The main influencing factors of different levels and duration of drought on summer maize were leaf biomass and root growth index. This showed that the results obtained by PCA analysis in this study were more reasonable. Therefore, this study proposed a comprehensive calculation method for evaluating the resilience of summer maize. The results of the PCA analysis were combined to quantitatively evaluate the resilience of summer maize. The evaluation results verified the previous hypothesis. The stronger the degree of drought, the weaker the resilience of summer maize. Under DR1, resilience was able to exceed the normal growth state during T3 and decreased below normal growth during T4. This also indicated that drought was able to affect the harvest period of summer maize even with continued rehydration. Under a range of light drought duration, the resilience of summer maize was improved. Under LDR1, resilience was lower than normal growth during T3 and increased beyond normal growth during T4, with a relative increase in yield. This indicated that summer maize needed some time and rehydration to recover. Even though summer maize recovered less well after rehydration, continued rehydration increased resilience and yield. Under LDR2, summer maize suffered yield losses despite higher resilience. Therefore, evaluation of summer maize resilience cannot only analyze growth characteristics after rehydration, but still needs to be combined with growth characteristics and yield during the sustained rehydration period. In this study, the osmotic metabolic processes of summer maize were not analyzed due to experimental constraints and will be added in future studies.

## 5 Conclusion

Based on field trials, the persistence effects of different levels and durations of drought on growth indicators and yield of summer maize were quantified, and a comprehensive evaluation method for summer maize resilience was proposed under different drought conditions. The results showed that the resilience of summer maize decreased as the drought level increased, and this effect was able to last until the harvesting period. However, under DR1, summer maize recovery was stronger at T3, but weakened at T4, while all growth indicators and yield decreased. In this process, drought reduced summer maize leaf biomass and root growth capacity in the 30-50 cm layer. In addition, a range of duration under light drought stress was able to improve the resilience of summer maize. Under SDR1, summer maize had less resilience during T3, but continued rehydration was able to improve resilience and yield. Therefore, a certain degree and duration of drought can improve the resilience and yield of summer maize under water deficit conditions. This study provides support for agricultural drought risk response and efficient water use. In the future, physiological changes will further be analysed under different drought conditions. Combined with crop growth models still needed to quantify the relationship between crop water consumption and resilience.

## Data availability statement

The raw data supporting the conclusions of this article will be made available by the authors, without undue reservation.

## Ethics statement

Written informed consent was obtained from the individual(s) for the publication of any potentially identifiable images or data included in this article.

## Author contributions

LJ and BW contributed to the conception and design of the study. DY confirmed and guided the study. LJ and SY carried out the experiments. LJ performed the statistical analysis and wrote the first draft of the manuscript. SZ, WB, and SY wrote sections of the manuscript. All authors contributed to the article and approved the submitted version.
